# 

**Published:** 1919-03-29

**Authors:** 


### MATRONS' AND SISTERS' APPOINTMENTS.

The Children's Hospital, Birkenhead.—Miss M. V. McNeece has been appointed ward sister. She was trained at the Victoria Hospital, Blackpool, and has since been staff nurse and acting sister at the Hospital, Port Sunlight.

The General Infirmary, Dewsbury.—Miss Ethel Barstow has been appointed sister. She was trained at Dewsbury and District General Infirmary. From 1909 to 1916 she was engaged in private nursing at Stoke Newington, in 1917 she joined the Q.A.I.M.N.S.R. at Salop, and in 1918 was sent to Italy. She holds the C.M.B., Commercial Road, London.

Raratonga Hospital, Cook Islands, Southern Pacific.—Miss F. Eileen L'Amie has been appointed matron. She was trained at the District Asylum, Inverness; the City Hospital, Edinburgh; the General Infirmary, Sunderland; and the Liverpool Maternity Hospital. She has been head nurse at the District Hospital, Coonamble, N.S.W., and has worked on the staff of the New South Wales Bush Nursing Association. Miss L'Amie holds the certificates of the Central Midwives Board, and is a member of the Australasian Trained Nurses' Association.

Hawkmoor Sanatorium, Bovey Tracey.—Miss Lillian G. Jackson has been appointed assistant matron. She was trained at the Royal Devon and Exeter Hospital, Exeter, and has served in the Territorial Force Nursing Service.

Bierley Hall Sanatorium, Bradford.—Miss E. A. Robinson has been appointed matron. She was trained at the Huddersfield Royal Infirmary, where she was later assistant matron, and has been matron of the General and Tuberculosis Hospital for Serbians in Corsica.

The Infirmary, Beckett Street, Leeds.—Miss Eliza Janet Williams has been appointed first assistant matron. She was trained at St. Thomas' Hospital, London, has been surgical, medical, and operating-theatre sister on a hospital ship under Queen Alexandra's Royal Naval Nursing Service Reserve, and has been sister at the Dreadnought Hospital, Greenwich.

General Hospital, Birmingham.—Miss Elizabeth Hatfield has been appointed sister. She was trained at the Royal Infirmary, Leicester. Previous appointments held were holiday sister, ward sister, and night sister of the Royal Infirmary, Leicester. She holds the C.M.B. Certificate, Queen Charlotte's Hospital, London.

Chase Farm School Infirmary, Enfield.—Miss Emily Dudley has been appointed nurse-matron. She was trained at Isleworth Infirmary, and has held appointments as school nurse, Bexhill-on-Sea, private nursing, staff nurse Enfield Cottage Hospital, and staff nurse and sister-in-charge of Convalescent Home (Red Cross work).

Royal National Hospital, Ventnor.—Miss Florence King has been appointed day sister. Trained at the General Hospital, Birmingham. She was afterwards sister-in-charge of a private nursing home, night sister and subsequently sister-in-charge of the Nevill Park Hospital, Tunbridge Wells.

Isolation Hospital, Stafford.—Miss Mary Barton has been appointed matron. She was trained at the Oakwell Joint Hospital, Birstall, near Leeds, and at West Bromwich and District Hospital. Her previous appointments were sister at the Borough Isolation Hospital, Stafford, and matron of Isolation Hospital, Tamworth.

Addenbrooke's Home of Recovery, Hunstanton.—Miss K. Newman has been appointed sister-in-charge. She was trained at the Hampstead General Hospital, has been staff nurse at the Cray Valley Hospital, Kent; night sister, sister of the medical women's and military wards at Addenbrooke's Hospital, Cambridge, where she has also taken assistant matron's holiday duty.

West Bromwich and District Hospital.—Mrs. G. M. E. Jones has been appointed sister-in-charge of the electrical massage and x-ray department and Miss F. Hamilton-Swinn ward sister. The former was trained, as Miss Leigh, at the Royal Eye Hospital and at Guy's Hospital, winning the Cazenove medal at the latter institution. Returning to nursing in 1915, she became sister-in-charge of Benefield Auxiliary Hospital, served for one year with the Almeric Paget Military Massage Corps, nursed at the 4th London General Hospital, was released for service in France, and became matron of a hospital under the French Red Cross; she later worked with the Scottish Women's Hospital Unit at Villers Cotterets. Mrs. Jones has also been night superintendent at the Throat Hospital, Golden Square. Miss Hamilton-Swinn was trained at St. Helens Hospital, has been sister at the White Cross Military Hospital, Warrington, and of the military ward at the Royal Infirmary, Derby.

